# Pathological laughing and psychotic disorder: the medical evaluation of the Joker

**DOI:** 10.1007/s13760-020-01332-3

**Published:** 2020-03-18

**Authors:** Alexis Demas, David Tillot

**Affiliations:** 1 Neurology Unit, Department of Neurology, Le Havre Hospital, 29 avenue Pierre Mendes, 76290 Le Havre Cedex, France; 2 Department of Anesthesiology, Universitary Hospital Charles Nicolles, 37 Boulevard Gambetta, 76000 Rouen, France

**Keywords:** Pathological laughing, Psychotic disorder, Traumatic brain injury

## Abstract

In the psychological thriller film *Joker,* released in 2019 and starring Joaquin Phoenix in the first role, another possible origin story for this iconic character is reported. Above all, it brings us medical elements for the understanding of the development of this complex character. Contrary to other interpretations, we discover a lonely, timid and uncharismatic man (Arthur Fleck). He seems to be suffering from psychobehavioral disorders and seems depressed. There is a strangeness in his behavior along with social withdrawal. He suffers from fits of laughter that occur at socially inappropriate times. He also suffers from psychotic symptoms with visual delusions. We learn through the film that he was a beaten child, psychologically and physically abused with severe traumatic brain injury (TBI). The uncontrollable outbursts of laughter, behavioral and psychotic disorders followed these elements. As a neurologist, I was intrigued by these symptoms. I have explored the neuropsychiatric symptoms complicating TBI from which he seems to suffer and which have been reported in the literature. We can assume that the Joker is suffering from neuropsychiatric sequelae related to childhood TBI involving the frontotemporal regions and, in particular, the lateral aspect of the left frontal lobe. The movie *Joker* has medical significance and covers social aspects of medicine and health care. First, it allows us to discuss whether psychotic disorder due to TBI should be considered a neurobiological syndrome. More broadly, albeit fictitious, it asks us about the management of patients with neuropsychiatric illness, which is a public health problem. It also reminds us that semiological descriptions of patients with neuropsychiatric disorders have served as inspiration for many authors.

## Introduction

The Joker, a supervillain created by Bill Finger, Bob Kane and Jerry Robinson in 1940, is one of the most iconic characters in popular culture. A symbol of madness, he is considered the antithesis of Batman. Throughout his appearances, whose form has evolved with the times, the Joker is depicted as a very fanciful and intelligent character, a master of crime. Instantly recognizable with his white skin, green hair, and bright red lips wearing a sardonic smile, these aspects are supposed to come from a fall into a tank of chemical waste. These physical changes and the pain would then drive him insane. This is one of the many origin stories that have been described since the initial description, and the one I remember from when I was a child. The Joker has subsequently been adapted in television series and in films by various actors. This can be a unique experience for an actor, and Heath Ledger also won the Best Performance in a Supporting Role award (*The Dark Knight*). Multiple actors cast as the Joker had similarities in the way they played this role in both the clothing and psychobehavioral aspects. Notably, eccentricity, antisociality and pure violence have been emphasized. Recently, the character was adapted into his own standalone film, *Joker*, in 2019 starring Joaquin Phoenix in the starring role. This psychological thriller film, directed and produced by Todd Phillips and co-written with Scott Silver, proposes another possible origin story for the character.

## Method

The film takes place in 1981 in Gotham City and follows Arthur Fleck, a failed stand-up comedian who lives with his mother. He officiates as a sign-spinning clown in a sordid business. The film is set against a backdrop of social misery and class struggle. Contrary to other interpretations, the main character appears to be a lonely, timid and uncharismatic man who spends his free time taking care of his mother who nicknamed him “Happy”. He seems to be suffering from psychobehavioral disorders and seems depressed with a relentless sense of morbid sadness. There is a strangeness in his behavior, and he seems to be socially withdrawn. Furthermore, there are impulsive and violent acts that are reprehensible by law. He appears very thin. Multiple large hematomas are visible on his chest and back after the scenes of aggression. We learn that he has been hospitalized in a psychiatric unit with violent outbursts. He continues to benefit from medical follow-up and treatment, including psychotropic drugs. Rapidly in the movie, one of the emblematic characteristics of the Joker appears: the laughter. These fits of laughter occur at socially inappropriate times (such as during the assault of a woman on the subway). These uncontrollable outbursts of laughter will be detrimental to him, and as a result of a series of assaults, he will sink into violence and crime. He also suffers from psychotic symptoms with visual delusions (imaginary love affair with his neighbor). We learn through the film that his mother has a psychotic disease with delusions (paranoid schizophrenia). At this point, we believe that he suffers from an inherited psychiatric illness with a schizotypal personality disorder. We then learn two fundamental elements. First, he was adopted. Therefore, no genetic link with the psychiatric pathology of the adoptive mother can be retained. However, this censoring effect does not exclude another family psychiatric pathology that we will not know. Second, he was a beaten child who was psychologically and physically abused with severe traumatic brain injury (TBI). The uncontrollable outbursts of laughter and the behavioral and psychotic disorders followed these elements. As a neurologist, I was intrigued by these symptoms, which leads us to propose a link between the after effects of the TBI he underwent and the various neuropsychiatric symptoms from which he seems to suffer.

## Results

Several studies have well established that TBI increases the risk of mood disorders, anxiety disorders, personality changes or substance use disorders [1]. Regarding the risk of developing psychosis, the data have been controversial. A meta-analysis of 172 studies published in 2011 revealed a significant association of approximately 60% between TBI and psychotic disorder (PD–TBI) [2]. Psychotic disorders can occur after both mild and moderate-to-severe TBI [3, 4]. A latency exists from the time of TBI to the onset of psychosis. Most cases occur within the first year [3]. However other studies report the development of psychosis after 5 or years after the event [4, 5]. The mean time from the TBI to the onset of psychosis appears to be between 4 and 5 years [6]. The contribution of TBI seems to be greater in subgroups of patients, notably among those with an inherited vulnerability to schizophrenia [7]. A study reported a highly significant tenfold increase in the risk for schizophrenia following head injury that occurred before the age of 10 years [8]. Lesions of the temporal and frontal lobes are most commonly implicated in psychosis after TBI [3, 9]. Differentiating PD–TBI from schizophrenia is difficult. A pattern of characteristics that differentiates or individualizes PD–TBI has been found [10]. The most common psychotic symptoms associated with PD–TBI are persecutory delusions and auditory hallucinations. Visual hallucinations, from which the Joker suffers, seem to be the second most common type of hallucinations. Negative symptoms are much less prominent. Cognitive impairments involve memory and executive functioning. A majority of patients improved with antipsychotic medication. These elements are compatible with the mental state of the Joker in the movie. Last but not the least, PD–TBI has been associated with more focal lesions in the frontal and temporal areas on structural and functional imaging, whereas the temporal areas have been implicated on electroencephalography (temporal slowing).

Another fascinating and precious neurological symptom must now be described: the pathological laughing. This can be described as uncontrollable episodes of laughing that are triggered by a stimulus that would not normally cause such an emotional response. It is classically associated with the pathological crying (pathological laughing and crying; PLC). PLC is an essential part of pseudobulbar palsy syndrome that is the consequence of bilateral lesions in the corticobulbar pathway, of which the etiologies are varied (stroke, Alzheimer’s disease, multiple sclerosis, etc.). A study revealed that the prevalence of PLC during the first year after TBI was 10.9% [11]. The release of cortical inhibition of the upper brainstem centers that integrate the motor activation patterns involved in laughing and crying would explain these aberrant emotional manifestations. PLC can also occur in patients with unilateral lesions that do not involve the motor or premotor area. Furthermore, PLC has been associated with anxiety disorder and focal frontal lobe lesions, especially in the lateral aspect of the left frontal lobe. Prefrontal dysfunction may also explain the observed association of PLC with the aggressive behavior. This is precious because one of the main features of the Joker thus becomes the witness of the injured cerebral zone.

The character has also suffered from psychotrauma, which may have led to post-traumatic stress disorder, with symptoms of their own (e.g. his responses in the subway, ultimately leading him to kill a few men). More importantly, Arthur displays quite severe social cognitive dysfunctioning. His theory of mind is clearly affected, as he is hardly able to read other people’s minds and reactions. At a certain point, he is trying to learn what makes other people laugh at a comedy club, making strange deductions in the process.

In light of all this, we can assume that the Joker is suffering from neuropsychiatric sequelae related to childhood TBI involving the frontotemporal regions and, in particular, the lateral aspect of the left frontal lobe. These elements occur in the context of urban violence and difficulties in accessing the medical care facility. The presence of a developmental disorder (pervasive developmental disorder like autism) associated with other co-morbid psychiatric disorders as well as pre-existing personality traits that have developed into a personality disorder are strongly suspected. Furthermore, undernutrition and vitamin C deficiency may have also been contributing factors to the development of multiple hematomas and psychiatric illnesses.

## Discussion

The movie *Joker* has medical significance. First, it allows us to discuss whether PD–TBI should be considered a neurobiological syndrome. More broadly, albeit fictitious, the movie asks us about the management of patients with neuropsychiatric illness, which is a public health problem. The boundaries between psychiatry and neurology are thin, and exchanges between these health professionals are essential.

Several symbols are highlighted in the movie. The choice of the clown as a symbol of tragicomedy is the most striking. As in Stephen King’s *It*, the clown becomes the counter-symbol to laughter and mystifies violence and tragedy. During the arrest of Arthur Fleck, the police car is hit by an ambulance driven by a supporter of the wave of violence. His release will lead to the scene where he will draw a smile with blood on his face and turn into the Joker. This is also another counter-symbol. The ambulance could represent the role of medicine in preventing this transformation and curing Arthur but by its hindrance or failure (the closing of medical practices due to budget cuts), it becomes the catalyst.

As a doctor, a surprising aspect of watching the film was to discover the human before the character, and going further, the patient. Joaquin Phoenix explained that he had observed people with neurological disorders to create his character and to forge the most realistic laughter possible, as close as possible to a real clinical pathology. The movie won the Golden Lion at Venice Film Festival 2019, and Joaquin Phoenix is up for an Oscar for Best Actor at the next Academy Awards.

Semiological descriptions of patients with neuropsychiatric disorders have served as inspiration for many authors. In his well-known children’s book *Alice’s Adventures in Wonderland* published in 1865, Lewis Carroll described that Alice felt her body growing both larger and smaller. Almost a century later (1955), the psychiatrist John Todd proposed the term Alice in Wonderland syndrome (AIWS) to cover a group of perceptual disorder symptoms characterized by distortions of visual perception (metamorphopsias), the body schema and the experience of time [12]. Main conditions include infectious diseases (Epstein-Barr virus encephalitis) and migraine. Lewis Carroll had migraines. His attacks were sometimes preceded by aural phenomena [13]. The bodily changes experienced by Alice might well have been inspired by body schema illusions the author had experienced himself.

The relationship between fiction, culture and medicine is, therefore, strong. Sometimes a fictional illness leads to medical reflection, such as the recent article (Greyscale—a mystery dermatologic disease on HBO’s *Game of Thrones*) about the dermatological disease suffered by characters in the popular television show *Game of Thrones* [14].

Medical advances have made it possible to explore the brain’s networks responsible for neuropsychiatric symptoms. For Alice, through the mirror of fictitious work based on real facts, we hope that this Joker will allow us to reflect on our care of patients with neuropsychiatric disorders.
